# Observations of Modified Polyps and Polyp Leaves in Sea Pens (Cnidaria: Octocorallia): The Cases of *Ptilella* and *Pennatula*

**DOI:** 10.1002/ece3.73868

**Published:** 2026-06-19

**Authors:** Bárbara de Moura Neves, Kathryn Murray, Rachelle Dove, Vonda E. Hayes, Carlos Daniel Pérez

**Affiliations:** ^1^ Department of Fisheries and Oceans Canada St. John's Canada; ^2^ Centro Acadêmico de Vitória Universidade Federal de Pernambuco Vitória de Santo Antão Brazil

## Abstract

Here we describe unusual morphological traits identified on colonies of the deep‐water sea pens *Ptilella grayi*, *Pennatula aculeata* and *Pennatula* sp. from the Northwest Atlantic, namely the presence of hypertrophied polyps, split polyp leaves, and autozooids budding on the surface of polyp leaves (as opposed to the edges). These observations are not in line with the species diagnoses, but there is no evidence to justify the colonies' identifications as different or new taxa. We also provide DNA sequence data for mitochondrial and nuclear markers and suggest a geographic range extension for *Ptilella grayi* into the NW Atlantic. Although we do not know the origin of these peculiarities, we hypothesize that given the rarity of this finding, predation could have resulted in the observed split polyp leaves in *Pt. grayi* and influence the development of hypertrophied polyps. Given that these sea pen taxa are some of the few taxa to display more than three types of polyps (i.e., polymorphism), we also discuss the hypothesis that hypertrophied polyps might differ from autozooids and represent an undescribed type of polyp. Nevertheless, future hypothesis‐driving studies are necessary to factually explain these observations.

## Graphical abstract

We describe unusual and mostly unreported morphological traits on colonies of the deep‐water sea pens *Ptilella* and *Pennatula* from the Northwest Atlantic, namely the presence of hypertrophied polyps, split polyp leaves, and autozooids budding on the surface of polyp leaves (as opposed to the edges).

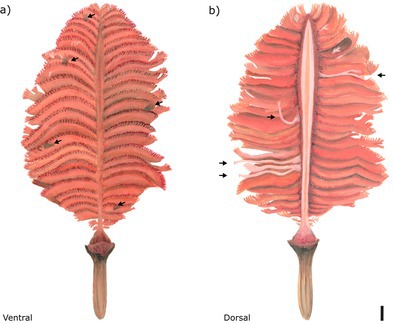

## 1 Introduction

Sea pens are colonial octocorals of worldwide distribution, being found from intertidal to abyssal zones (Williams 2011). They are distinctive from other octocorals because they possess a muscular peduncle that anchors the colony in soft (or even hard) sediments. Colonial organization in sea pens is also differentiated, as in many species polyps are found distributed along lateral expansions called polyp leaves (Williams 1995).

Sea pens are polymorphic octocorals, where at least three different types of polyps can be found in the same colony: oozoid, siphonozooids, and autozooids (Bayer et al. 1983; Williams 1995). Some taxa present a fourth and fifth type of polyps, the mesozooids and acrozooids (Williams et al. 2012). Autozooids are used for food capture, digestion, and reproduction; siphonozooids and mesozooids are believed to mostly play a role in water exchange through the colony, while acrozooids appear to have a reproductive role (Hickson 1916; Williams et al. 2012). Within a colony, autozooids do not vary significantly in morphology or overall size, with slight deviations in size related to location in the colony/polyp leaf or life stage. Similarly, polyp leaves vary in size depending on position on the colony and ontogeny (Hickson 1916; Williams 1995), but to our knowledge, substantial intracolony morphological variation has not been reported.

The sea pens *Ptilella grandis* (Ehrenberg, 1834) (until recently known as *Pennatula grandis*) (García‐Cárdenas et al. 2019) and *Pennatula aculeata* Danielssen, 1860 are two common species in the Northwest Atlantic, often reported as fishing bycatch from mean depths ranging between 182 and 1404 m (Wareham and Edinger 2007). The co‐generic sea pen *Ptilella grayi* was described in 2019 based on specimens from the Rockall Bank, NE Atlantic (García‐Cárdenas et al. 2019), and not yet reported in the NW Atlantic. *Ptilella grandis* and *Pt. grayi* are morphologically similar and differ mostly based on the size of sclerites and gene substitutions (García‐Cárdenas et al. 2019). Colonies of *Pt. grandis*, *Pt. grayi* and *P. aculeata* have well‐developed polyp leaves and possess mesozooids in addition to autozooids and siphonozooids (Williams 1995; García‐Cárdenas et al. 2019).

During an expedition to the Labrador Sea (Northwest Atlantic), a colony of *Ptilella* was collected using a remotely operated vehicle (ROV). Immediately after collection, we identified the colony as *Pt. grandis*, given the similarity with the species, which is commonly found in the region. Nevertheless, we noticed that this colony exhibited certain unexpected features namely hypertrophied polyps (elongated polyps), split polyp leaves (i.e., divided into two or more parts), and autozooids budding at the surface of some polyp leaves, none of which are diagnostic traits of *Ptilella*. Hypertrophied polyps are visually comparable to autozooids, except that they are extremely long, rare, and appear randomly distributed on the colony (see the Material and Methods section for a detailed definition). Puzzled by these findings, we performed molecular analyses for three genes (two mitochondrial and one nuclear) in this specimen to confirm species identification. Our results showed that the specimen in fact matches *Pt. grayi*, which constitutes its first report in the NW Atlantic, outside of its type‐location in the NE Atlantic (detailed characterization will follow in a different publication). After this discovery, inspection of two additional colonies of *Ptilella* from a different location in the Labrador Sea (~430 km apart), as well as the Laurentian Channel (NW Atlantic) yielded similar observations.

Later, we identified similar morphological peculiarities (hypertrophied polyps and polyp leaf splits) on specimens of *P. aculeata* and a sea pen morphologically similar to *P. aculeata* identified as *Pennatula* sp., also collected near/from the Laurentian Channel. Here we describe these observations and hypothesize on their occurrence. Our goal is to generate awareness about the presence of these characters in additional colonies and to stimulate further studies to investigate their origins, frequencies, and potential roles in a colony.

## 2 Material and Methods

### 2.1 Sample Collection and Observations

**FIGURE 1:**
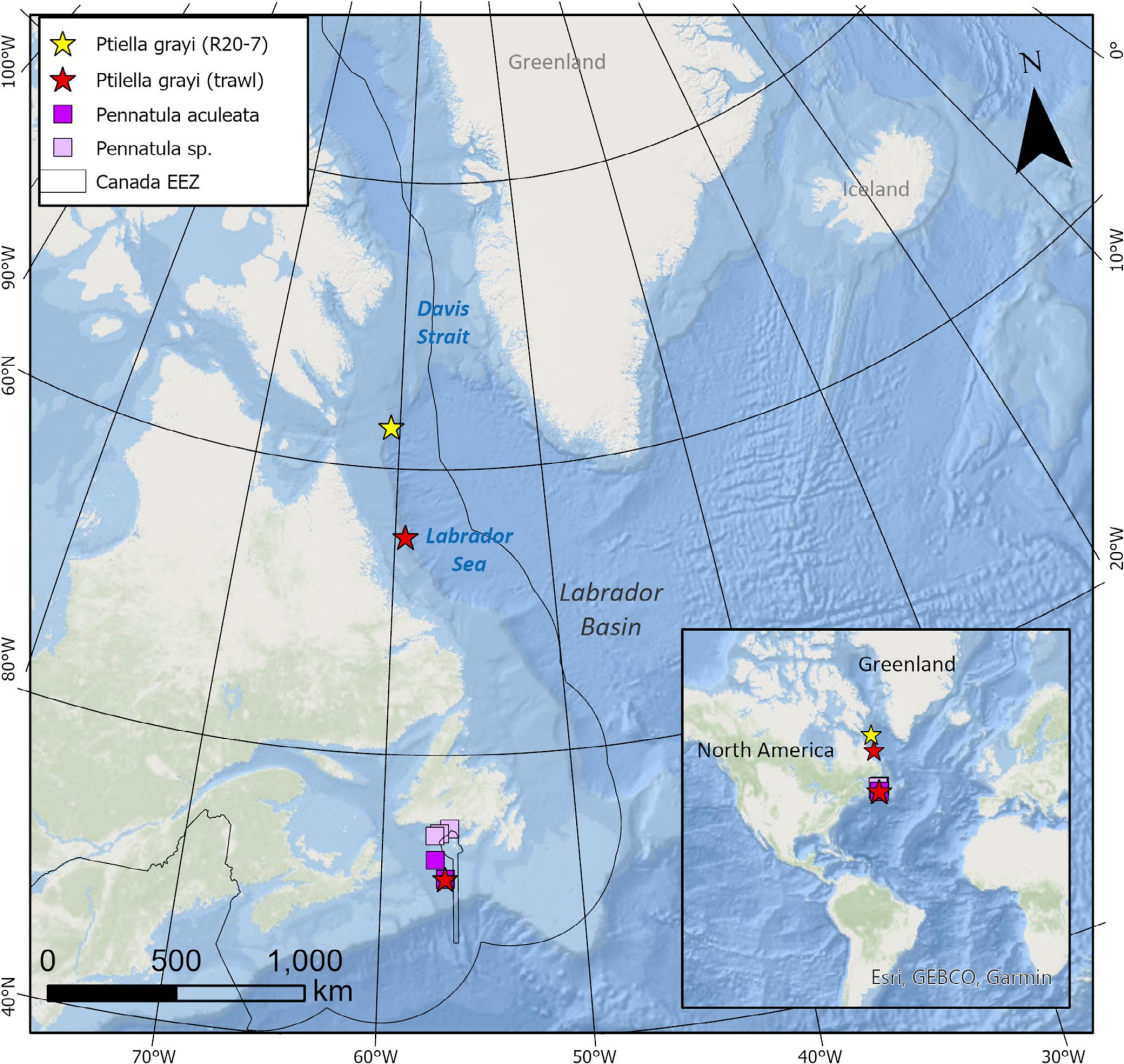
Location of the sea pen samples included in this study (Northwest Atlantic).

The first *Ptilella* colony (R20‐7) was collected using the ASTRID ROV aboard CCGS *Amundsen* on July 27th, 2021, at Hatton Basin (northern Labrador Sea), NW Atlantic (61.43 N, 60.64 W) at 698 m deep (Figure 1). Two polyp leaves were frozen at −80°C for a different project, a small fragment in 100% ethanol for DNA barcoding (see below), and the remaining (most of the colony) preserved in 70% ethanol. Colony R20‐7 had a scale worm on it, and DNA sequencing was conducted for both the sea pen and the worm.

Two *Ptilella* colonies (colony 14706, set 39‐222‐19 and colony 15934, set 77‐21‐20) were collected during Fisheries and Oceans Canada (DFO, Newfoundland and Labrador Region) bottom trawl surveys in October 2021 and April 2022, respectively (Table 1). Colony 14706 was collected inside the Hopedale Saddle Marine Refuge offshore Labrador (57.62 N, 59.33 W) from a mean depth of 783 m (Figure 1). Colony 15934 was collected inside the Laurentian Channel Marine Protected Area (45.65 N, 56.80 W) from a mean depth of 359 m. The specimens were frozen at −20°C (as per survey protocol) and preserved in 70% ethanol afterwards.

**TABLE 1:** Metadata of samples included in this study including Canadian Museum of Nature catalog number (CMN Cat #). For trawl samples, depth is mean depth; latitude and longitude are start positions. B temp = bottom temperature.

Sample ID	CMN Cat #	Species	Gear	Year	Depth (m)	B temp (°C)	Latitude	Longitude
R20‐7	CMNI 2026–0009	*Ptilella grayi*	ROV	2021	698	4.3	61.4362	−60.6416
14706	CMNI 2026–0010	*Ptilella grayi*	Campelen trawl	2021	783	4.2	57.6180	−59.3280
15934	CMNI 2026–0011	*Ptilella grayi*	Campelen trawl	2022	359	6.2	45.6535	−56.7952
15794‐1	CMNI 2026–0012	*Pennatula* sp.	Campelen trawl	2024	200	6.6	46.3290	−57.3033
15794‐2	CMNI 2026–0013	*Pennatula aculeata*	Campelen trawl	2024	200	6.6	46.3290	−57.3033
15798	CMNI 2026–0014	*Pennatula* sp.	Campelen trawl	2024	262	6.7	47.2568	−57.0833
15791	CMNI 2026–0015	*Pennatula* sp.	Campelen trawl	2024	264	6.6	47.4205	−56.5617
15657	CMNI 2026–0016	*Pennatula* sp.	Campelen trawl	2025	240	6.3	47.1773	−57.3228
15942‐1	CMNI 2026–0017	*Pennatula aculeata*	Campelen trawl	2022	359	6.2	45.6535	−56.7952
15942‐2	CMNI 2026–0018	*Pennatula aculeata*	Campelen trawl	2022	359	6.2	45.6535	−56.7952

Colonies of *P. aculeata* (*n* = 3) and *Pennatula* sp. (*n* = 4) were also collected during DFO bottom trawl surveys. They were collected from multiple sites south of Newfoundland (Figure 1) from mean depths of ~200–359 m in April of 2022, 2024, and 2025. These specimens were initially frozen at −20°C and later preserved in 70% ethanol. Colonies identified as *Pennatula* sp. are morphologically similar to *P. aculeata* but do not match the full species description and could represent a different, still unidentified species. No DNA sequencing was conducted for these sea pens, and identification was based on morphology. Sea pen colonies were deposited at the Canadian Museum of Nature under catalog numbers CMNI 2026‐0009‐CMNI 2026‐0018 (Table 1).

Both ventral and dorsal sides of the colonies were inspected. Three main oddities were identified (although not in all taxa and specimens) and the following terms will be used to describe these features hereafter: (1) polyp hypertrophy, (2) polyp leaf split, and (3) autozooid budding on the surface of polyp leaves. Here we define hypertrophied polyps as either (i) an elongated single polyp that raises from/near a polyp leaf or (ii) a “branch” that raises from/near a polyp leaf, and which terminates in two or more polyps. Hypertrophied polyps resemble autozooids and can be as long as a polyp leaf. Because the anatomy of the hypertrophied polyps has not been investigated due to their preservation mode, we refer to them as polyps, not as autozooids. Polyp leaves were considered split if a leaf was partially divided into two or more parts through the length of the leaf, or if a split took place at the base of the leaf.

Polyp leaf pairs were counted and mapped relative to their position from a dorsal perspective into left (L) and right (R). We considered their position in ascending order from the distal to proximal portion of the rachis (i.e., polyp leaf pair 1—PL 1—at the tip of the colony). Given the rarity of the hypertrophied polyps, we opted to not dissect them. When autozooids were observed growing on the surface of polyp leaves, we classified them as either autozooid buds (budding) when they were isolated polyps, or as autozooid bundles, when they were growing together.

### 2.2 Molecular Analysis

DNA was extracted from ethanol‐preserved *Ptilella* colonies and scale worm tissue samples using QIAgen DNeasy Blood and Tissue Kit, with an initial overnight incubation with Proteinase K, and stored at −20°C in the elution buffer provided, following the manufacturer's protocol. For the sea pens, three mitochondrial genes were targeted: *mtMutS*, the cytochrome oxidase I (*COI*) and NADH dehydrogenase‐2 (*ND2*), and one nuclear gene: the *28S* nuclear ribosomal DNA (*28S*). Polymerase chain reactions (PCRs) were performed using the following primers: (1) ND42599F (France and Hoover 2002) and mut3458R (Sánchez et al. 2003) for the *mtMutS* gene (McFadden et al. 2011); (2) 16S647F and ND21418R (McFadden et al. 2004) for *ND2*; (3) COII8068F (McFadden et al. 2004) and COIOCTR for COI (France and Hoover 2002), and (4) *28S*‐Far and *28S*‐Rab (McFadden and Van Ofwegen 2012) for the *28S* gene.

The PCR amplification of *mtMutS*, *ND2*, *COI*, and *28S* rDNA gene sequences was conducted using 12.5 μL of Green Dream Master Mix, 1 μL of template DNA, 0.5 μL of forward primer, 0.5 μL of reverse primer, and 10.5 μL of water. Thermocycling was run as follows: 3 min of initial denaturation at 95°C, followed by 35 (*mtMutS* sample 14706, *COI*) or 40 (*mtMutS* other samples, *28S*) cycles at 95°C for 30 s, 30 s at annealing temperatures of 48°C for *mtMutS* and *28S*, 50°C for *ND2*, and 54°C for *COI*, then 65 s for *mtMutS* and *28S* and 30 s for *ND2* at an extension temperature of 72°C, ending with a final elongation at 72°C for 4 min.

For the scale worm, we targeted two mitochondrial genes, *COI* and ribosomal DNA (*16S*). PCRs were performed using gene‐specific sets of primers: (1) LCO1490 and HCO2198 (Folmer et al. 1994) for *COI*; (2) 16S‐arL and 16S‐brH (Palumbi 1996) for the *16S* gene.

The PCR amplification of *COI* and *16S* rDNA gene sequences was conducted using the same reagents and amounts described above for the sea pens. Thermocycling was run as follows: 5 min of initial denaturation at 95°C, followed by 25 cycles at 95°C for 30 s, 30 s at annealing temperatures of 42°C, then 30 s at an extension temperature of 72°C, ending with a final elongation at 72°C for 5 min. PCR products were cleaned using Agencourt AMPure XP Beads (Beckman Coulter, Brea, CA, USA) following the manufacturer's protocol and sent to The Centre for Applied Genomics (Toronto, Canada) for Sanger sequencing. Sequences were visualized and aligned using Geneious Prime 2025.0.3. Obtained sequences have been deposited in GenBank under accession numbers PX667528‐PX927506. Sequences were compared to available sequences on GenBank using BLAST's (blastn) default parameters on January 20th, 2026, and May 22nd, 2026.

## 3 Results

### 3.1 Molecular Analysis

Molecular analysis of *mtMutS* and *ND2* for sample R20‐7 and *mtMutS* and *COI* for samples 14706 and 15934 yielded 100% matches to a *Ptilella grayi* paratype's sequence (MK603846) (García‐Cárdenas et al. 2019) and sequences from two other *Pt. grayi* specimens (López‐González 2022) from the NE Atlantic (Table 2). Similarly, *28S* sequences matched *Pt. grayi* holotype's (MK882495) and paratype's (MK603853) sequences. A separate study on the geographic extension range of *Pt. grayi* into the NW Atlantic is currently ongoing.

**TABLE 2:** List of specimens included in this study, associated marker, accession numbers and their closest GenBank matches, with percent identity (% ident) and query coverage (Qcover) as ratio of match to search length in base pairs.

Sample ID	Marker/accession number	Match accession number	% Ident	Qcover	Match taxon	Match references
R20‐7	*mtMutS* PZ399220	MK603846	100%	100%	*Ptilella grayi*	García‐Cárdenas et al. (2019)
		MW862999	100%	100%	*Ptilella grayi*	López‐González (2022)
	*ND2* PX907412	MW863008	100%	98%	*Ptilella grayi*	López‐González (2022)
		MW863009	100%	98%	*Ptilella grayi*	López‐González (2022)
		MW863010	100%	98%	*Ptilella grayi*	López‐González (2022)
	*28S* PX903727	MK882495	100%	92%	*Ptilella grayi*	García‐Cárdenas et al. (2019)
		MK603853	99.83%	92%	*Ptilella grayi*	García‐Cárdenas et al. (2019)
14706	*mtMutS* PZ399221	MK603846	100%	100%	*Ptilella grayi*	García‐Cárdenas et al. (2019)
		MW862999	100%	100%	*Ptilella grayi*	López‐González (2022)
	*COI* PZ379259	MK603856	100%	100%	*Ptilella grayi*	García‐Cárdenas et al. (2019)
		MW858344	100%	100%	*Ptilella grayi*	López‐González (2022)
		MK882497	100%	100%	*Ptilella grayi*	García‐Cárdenas et al. (2019)
	*28S* PZ379057	MK882495	100%	92%	*Ptilella grayi*	García‐Cárdenas et al. (2019)
		MK603853	99.83%	92%	*Ptilella grayi*	García‐Cárdenas et al. (2019)
15934	*mtMutS* PZ399222	MK603846	100%	100%	*Ptilella grayi*	García‐Cárdenas et al. (2019)
		MW862999	100%	100%	*Ptilella grayi*	López‐González (2022)
	*COI* PZ379258	MK603856	100%	100%	*Ptilella grayi*	García‐Cárdenas et al. (2019)
		MW858344	100%	100%	*Ptilella grayi*	López‐González (2022)
		MK882497	100%	100%	*Ptilella grayi*	García‐Cárdenas et al. (2019)
	*28S* PZ379056	MK603853	100%	92%	*Ptilella grayi*	García‐Cárdenas et al. (2019)
		MW862996	100%	92%	*Ptilella grayi*	López‐González (2022)

The scale worm sample (GenBank accession numbers PX667528 and PX927506) had a strong match to *Neopolynoe acanellae* (Verrill, 1882) (family Polynoidae): 99.67% for *COI* (OL792628) and 100% for *16S* (MN653087).

### 3.2 General Observations

Anomalies were observed in all examined colonies (Table 3). Hypertrophied polyps were present in *Pt. grayi* samples R20‐7 (*n* = 5), 14706 (*n* = 2), and 15934 (*n* = 3), *P. aculeata* (3 colonies, *n* = 1–24), and *Pennatula* sp. (4 colonies, *n* = 2–10) (Table 3). Polyp leaf splits were observed in *Pt. grayi* and *Pennatula* spp., although in the former they appear more along the polyp leaves, while in *Pennatula* spp. they often took place at the base of the polyp leaf (Table 3). A total of ten split polyp leaves were counted in sample R20‐7, four in sample 14706, and two in sample 15934. Polyps budding on the surface of polyp leaves were only observed in *Pt. grayi*. These observations are described in detail in the next sections.

**TABLE 3:** Observations of hypertrophied polyps, autozooid budding at the surface of polyp leaves, and split polyp leaves on *Ptilella grayi* (colonies R20‐7, 14706 and 15934), *Pennatula aculeata* (colonies 15794‐2, 15942‐1, 15942‐2), and *Pennatula* sp. (colonies 15794‐1, 15798, 15791, 15657). PL, polyp leaf.

Species	Colony	Location	Left polyp leaf	Right polyp leaf	Notes
*Ptilella grayi*	R20‐7	1	Standard	Standard	
		2	Autozooid bundle (5 polyps)	Standard	
		3	Standard	Standard	
		4	Standard	Split PL + autozooid bundle with 2 polyps, same as #12 on left	
		5	Standard	Standard	
		6	Standard	Autozooid bud on PL (upper surface)	
		7	Standard	Autozooid bundle (upper surface) with 4 polyps	
		8	Standard	Autozooid bundle (upper surface) with 7 polyps +1 polyp bud (upper surface). Budding (lower surface): 1 autozooid growing separate and 2 united at base.	
		9	Split PL	Hypertrophied polyp (*n* = 1). Autozooid budding on PL with two polyps (lower surface)	Hypertrophied polyp also splits into two
		10	Split PL	Split PL	
		11	Standard	Autozooid budding on PL with 2 polyps that are not connected to each other (lower surface)	
		12	Hypertrophied polyp coming from row of mesozooids, 1.3 cm long + split PL	Split PL	
		13	Standard	Standard	
		14	Hypertrophied polyp (*n* = 1, 4 cm long)	Split PL	Double split
		15	Standard	Standard	
		16	Autozooid bud on PL (upper surface)	Standard	
		17	Standard	Split PL	Double split
		18	Split PL	Standard	
		19	Hypertrophied polyp (*n* = 1, 5 cm long), no PL	Standard	
		20	Split PL + hypertrophied polyp (*n* = 1, 5 cm long). Polyp leaf arm with 5 autozooids.	Standard	
		21–28	Standard	Standard	
	14706	1–3	Standard	Standard	
		4	Modified PL/hypertrophied polyps (2 polyps)	Standard	
		5	Split PL	Standard	First part of split with three autozooids
		6	Split PL	Standard	
		7	Standard	Standard	
		8	Standard	Autozooid budding on PL (lower surface)	
		9–11	Standard	Standard	
		12	Autozooid budding on PL (Figure 5B)	Standard	
		13	Standard	Hypertrophied polyp (*n* = 2)	
		14–15	Standard	Standard	
		16	Hypertrophied polyp	Standard	
		17–20	Standard	Standard	
		21	Standard	PL split	Both sides of split have autozooids
		22	Standard	3 autozooids budding at base of PL (upper surface) which are not connected to each other	
		23	Standard	PL split	First part with three autozooids
		24	Standard	One autozooid slightly separated from rest of PL.	
		25–37	Standard	Standard	
*Ptilella grayi*	15934	1–3	Standard	Standard	
		4	Autozooid budding on rachis	Standard	Budding occurs just below the base of L4: two polyps, attached to each other.
		5	Standard	Standard	
		6	Standard	Split PL	Portion split from polyp leaf contains 4 autozooids.
		7	Standard	Split PL	The portion split from the leaf has 7 polyps. One single polyp Splitting off the margin of the polyp leaf adjacent to the first split.
		8	Standard	Standard	
		9	Autozooid bud on PL	Standard	Autozooid bud in middle of polyp leaf on lower surface.
		10	Standard	Standard	
		11	Standard	Hypertrophied polyp (1 cm)	Hypertrophied polyp split from PL margin, ~0.5 cm from base of polyp leaf.
		12–17	Standard	Standard	
		18	Hypertrophied polyps (1.5 cm)		Hypertrophied branch from the margin of the polyp leaf, along the mesozooids, terminating in two polyps
		19–44	Standard	Standard	
*Pennatula* sp.	15794‐1	1–11	Standard	Standard	
		12	Standard	Hypertrophied polyp (1.5 cm)	
		13	Standard	Standard	
		14	Hypertrophied polyp (2 cm)	Standard	
		15	Standard	Hypertrophied polyp (0.9 cm)	
		16–23	Standard	Standard	
		24	Standard	Hypertrophied polyp (2.5 cm)	
		25	Hypertrophied polyp (2.5 cm)	Hypertrophied polyp (1.6 cm)	
		26	Hypertrophied polyp (2 cm)	Standard	
		27–49	Standard	Standard	
*Pennatula aculeata*	15794‐2	1–16	Standard	Standard	
		17	Standard	Hypertrophied polyp (1.3 cm)	
		18–19	Standard	Standard	
		20	Standard	Hypertrophied polyp (1.7 cm)	
		21–38	Standard	Standard	
*Pennatula* sp.	15798	1–13	Standard	Standard	
		14	Standard	Hypertrophied polyp (1 cm)	
		15	Hypertrophied polyp (1.5 cm)	Standard	
		16–17	Standard	Standard	
		18	Standard	Hypertrophied polyp (~1.3 cm)	
		19–35	Standard	Standard	
*Pennatula* sp.	15791	1–5	Standard	Standard	
		6	Standard	Hypertrophied polyps	PL splits into two, then further splits into a hypertrophied Polyp and a hypertrophy with two polyps
		7–15	Standard	Standard	
		16	Standard	Hypertrophied polyp (1.3 cm)	Hypertrophied polyp splits from PL
		17–41	Standard	Standard	
*Pennatula* sp.	15657	1–18	Standard	Standard	
		19	Hypertrophied polyp (2.8 cm)	Standard	
		20	Hypertrophied polyp (2.1 cm)	Standard	
		21	Hypertrophied polyp (2.2 cm)	Hypertrophied polyp (1.9 cm)	
		22	Standard	Hypertrophied polyp (2.5 cm)	
		23	Hypertrophied polyp (1.7 cm)	Hypertrophied polyp (1.9 cm)	
		24	Hypertrophied polyp (1.5 cm)	Standard	
		25	Standard	Hypertrophied polyp (1.5 cm)	
		26–28	Standard	Standard	
		29	Standard	Hypertrophied polyp (0.7 cm)	
		30–34	Standard	Standard	
*Pennatula aculeata*	15942‐1	1–5	Standard	Standard	
		6	Standard	Hypertrophied polyp (2.5 cm)	
		7	Standard	Standard	
		8	Hypertrophied polyp (1.9 cm)	Standard	
		9–11	Standard	Standard	
		12	Hypertrophied polyp (0.7 cm, broken)	Hypertrophied polyp (1.9 cm)	
		13	Hypertrophied polyp (3.4 cm)	Standard	
		14	hypertrophied polyp (3.4 cm)	Hypertrophied polyp (3.0 cm)	
		15	Hypertrophied polyp (3.5 cm)	Standard	
		16	Hypertrophied polyp (3.4 cm)	Hypertrophied polyp (1.6 cm)	
		17	Standard	Standard	
		18	Hypertrophied polyp (3.2 cm)	Standard	
		19	Hypertrophied polyp (1.4 cm)	Hypertrophied polyp (3.2 cm)	
		20	Standard	Hypertrophied polyp (3.2 cm)	
		21	Standard	Standard	
		22	Hypertrophied polyp (1.0 cm)	Standard	
		23	Standard	Standard	Hypertrophied polyp attached at base to PL
		24	Hypertrophied polyp (0.7 cm)	Standard	
		25	Standard	Standard	
		26	Standard	Hypertrophied polyp (3 cm)	
		27	Hypertrophied polyp (0.9 cm, broken)	Standard	
		28–35	Standard	Standard	
		36	Standard	Hypertrophied polyp (1.5 cm)	
		37–38	Standard	Standard	
		39	Hypertrophied polyp (0.8 cm, broken)	Standard	
		40	Hypertrophied polyp (0.9 cm)	Standard	
		41	Standard	Hypertrophied polyp (1 cm)	
		42	Hypertrophied polyp (0.3 cm, broken)	Standard	
		43	Hypertrophied polyp (0.6 cm)	Standard	
		44–49	Standard	Standard	
*Pennatula aculeata*	15942‐2	1–5	Standard	Standard	
		6	Standard	Atrophied PL	3 polyps on PL.
		7	standard	Atrophied PL	9 polyps on PL
		8	Split PL with hypertrophied polyp (1.3 cm)	Standard	PL splits 0.3 cm from base
		9–43	Standard	Standard	

### 3.3 *Ptilella grayi* (R20‐7)

#### 3.3.1 General Morphology

**FIGURE 2:**
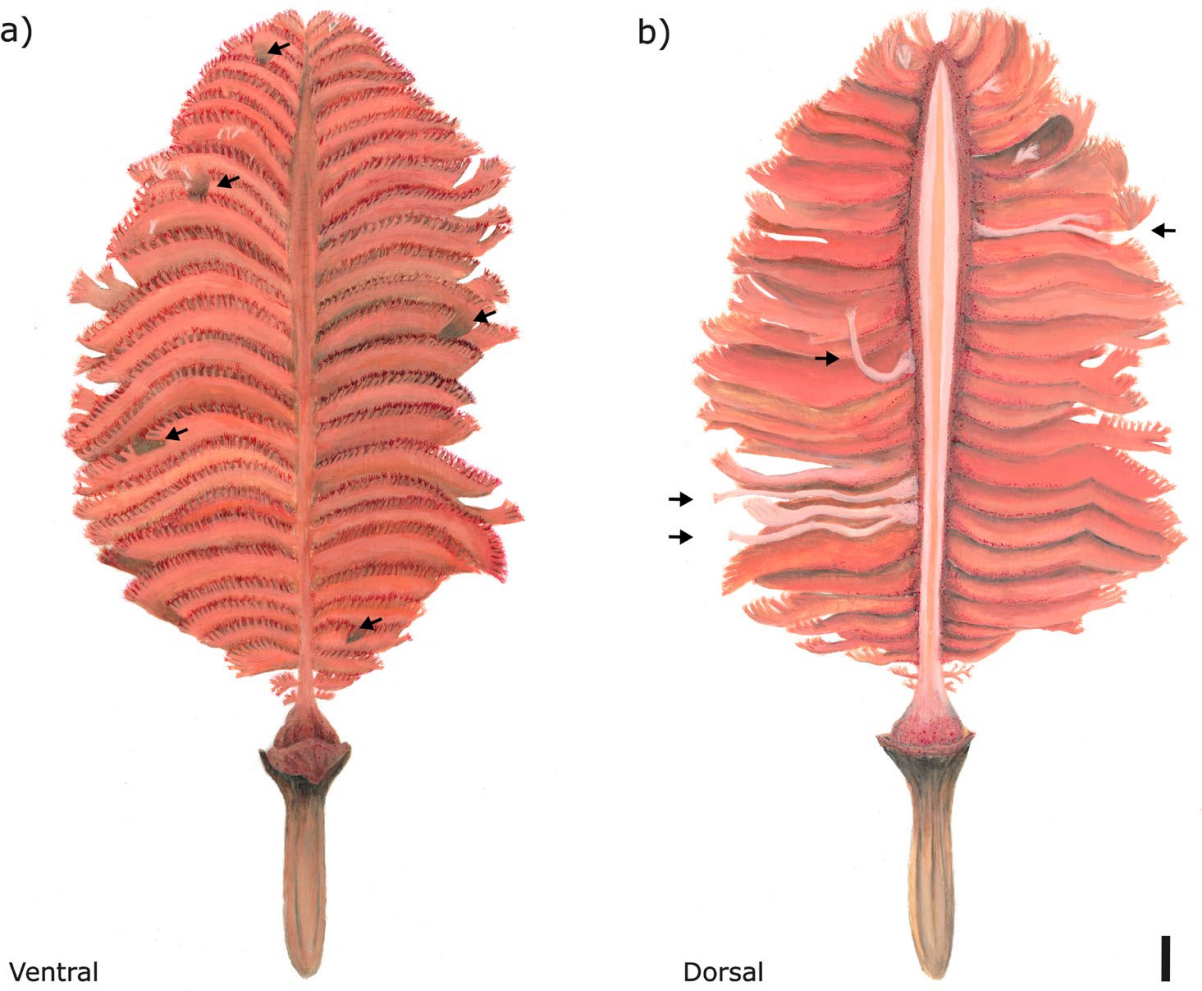
Illustration of ventral and dorsal sides of *Ptilella grayi* (sample R20‐7). Arrows in (a) point to split polyp leaves, and in (b) to hypertrophied polyps. Scale bar: 2 cm. See details in Figures 3 and 4. Illustrations by Kathryn Murray.

**FIGURE 3:**
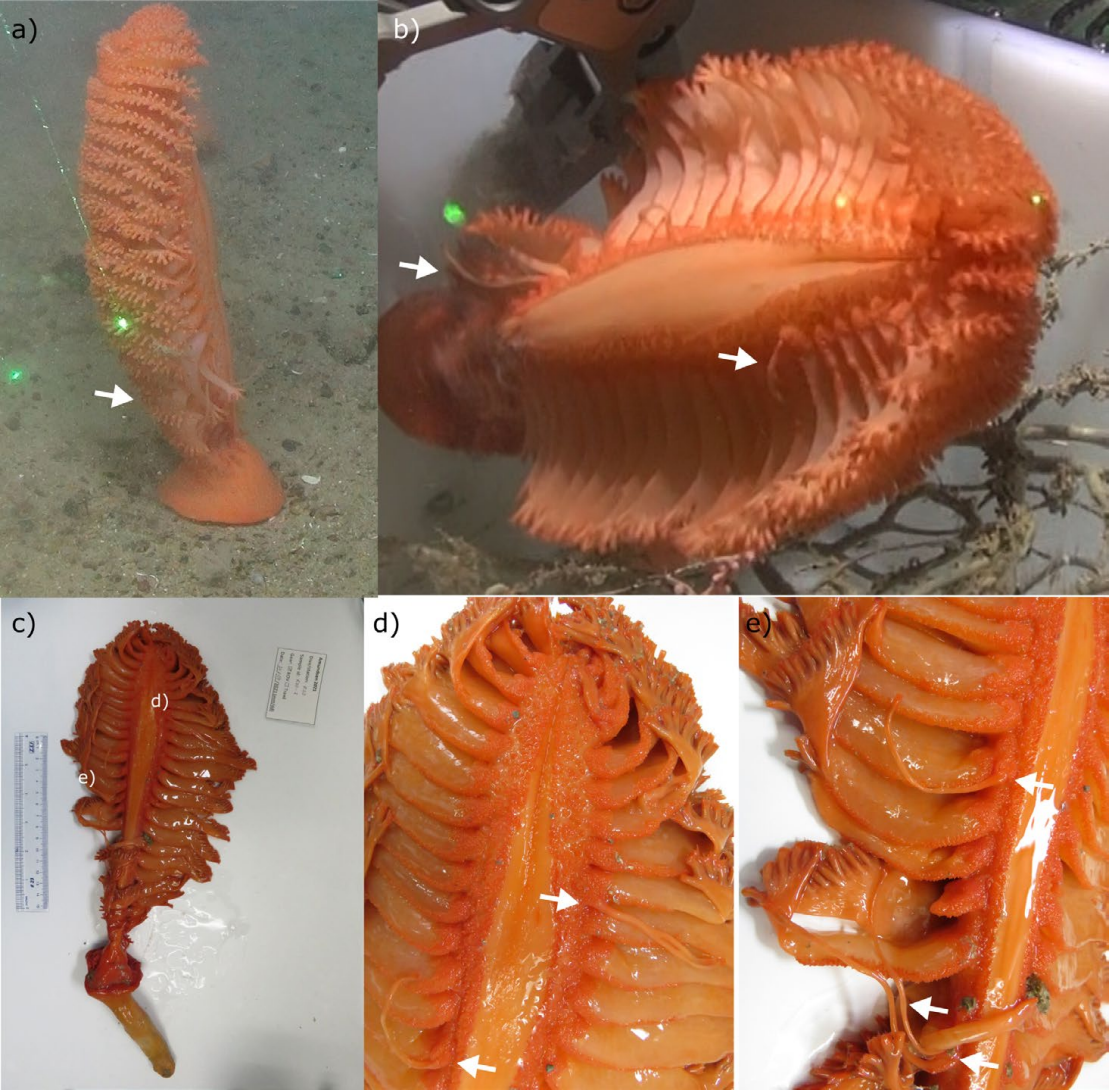
Colony of *Ptilella grayi* (sample R20‐7) in situ (a, b), showing hypertrophied polyps (arrows in a and b), and immediately after collection (c–e) also highlighting hypertrophied polyps (arrows in d) and (e).

Colony R20‐7 is 26.5 cm in total length, with a peduncle 12.6 cm in length (Figure 2, Figure 3). It has 28 polyp leaves, which are symmetrically opposed between polyp leaves 1 and 18, then alternate.

#### 3.3.2 Polyp Hypertrophy

**FIGURE 4:**
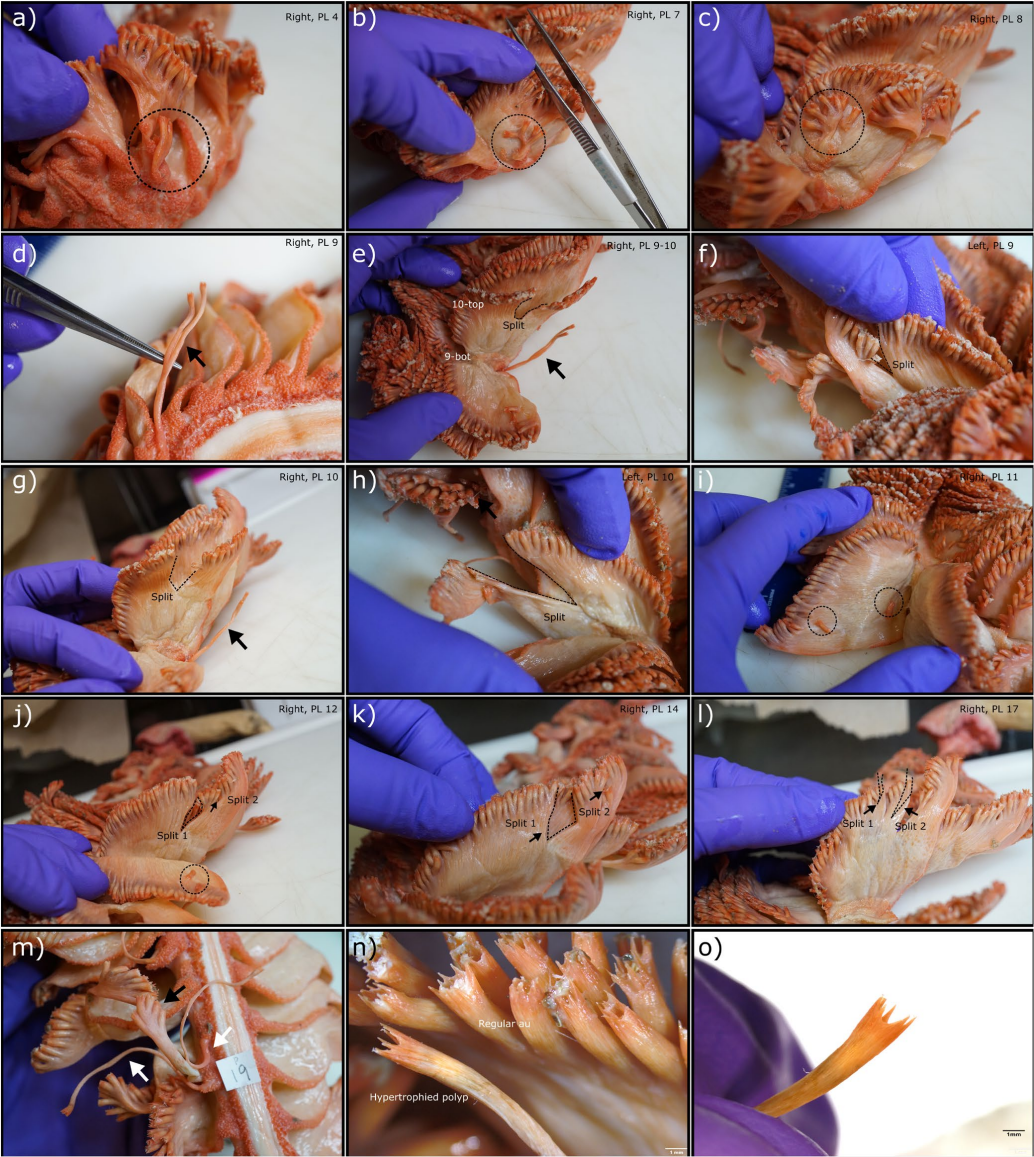
Close‐up of atypical features found on *Ptilella grayi* (sample R20‐7). Polyp leaf splits are emphasized by black dashed lines. Autozooid buddings on dorsal side of some polyp leaves are identified by dashed circles. Au, autozooid; PL, polyp leaf.

Four extremely long polyps (40–50 mm; i.e., hypertrophied polyps) were identified on colony R20‐7, three on the left side and one on the right side (Figure 2, Figure 4). These polyps present the same color as autozooids, a crown with eight teeth, and standard three‐flanged sclerites (Figure 4O). The one on the right side originated from the base of polyp leaf R9 and was almost the same length as the whole corresponding polyp leaf, terminating in two polyps (Figure 2 and Figure 4D). The three on the left side originated either from polyp leaf L14, L20 or in the case of L19, did not have a leaf present (Figure 2 and Figure 4M). These polyps follow the length of polyp leaves but are not part of the polyp leaves. Given that in this colony autozooids are around 6 mm in length, hypertrophied polyps are at least eight times longer than autozooids (Figure 4N,O).

#### 3.3.3 Polyp Leaf Split

A total of ten polyp leaves were split in two (or more), five on each side of colony R20‐7 (but not symmetrically, Table 3). There was no clear pattern in their arrangement. On the left side, polyp leaves L9, L10, L12, L18, and L20 were split, while on the right side they were R4, R10, R12, R14, and R17 (Figure 4). The split in L20 is characterized by two portions where one portion appears as a typical polyp leaf and the other as an arm with five autozooids (Figure 4M). Polyp leaves R14 and R17 had two splits (Figure 4). The splits' edges are smooth and both autozooids and whole polyp leaf have a typical appearance.

#### 3.3.4 Autozooid Budding on Surface of Polyp Leaves

Some polyp leaves had autozooids growing from the middle of the polyp leaf, rather than from the edge (Figure 4), as is usual in sea pens with polyp leaves. This was only observed on polyp leaves R6, R8, R9 in colony R20‐7 (Figure 4B,C,I,J). This “extra growth” consisted of 1–8 polyps growing sometimes as a bundle (Figure 4B,C).

#### 3.3.5 *Associated* Neopolynoe Acanellae *(Scale Worm)*

The scale worm was ~5 cm in length and found on specimen R20‐7.

### 3.4 *Ptilella grayi* (Colonies 14706 and 15934)

#### 3.4.1 General Morphology

**FIGURE 5:**
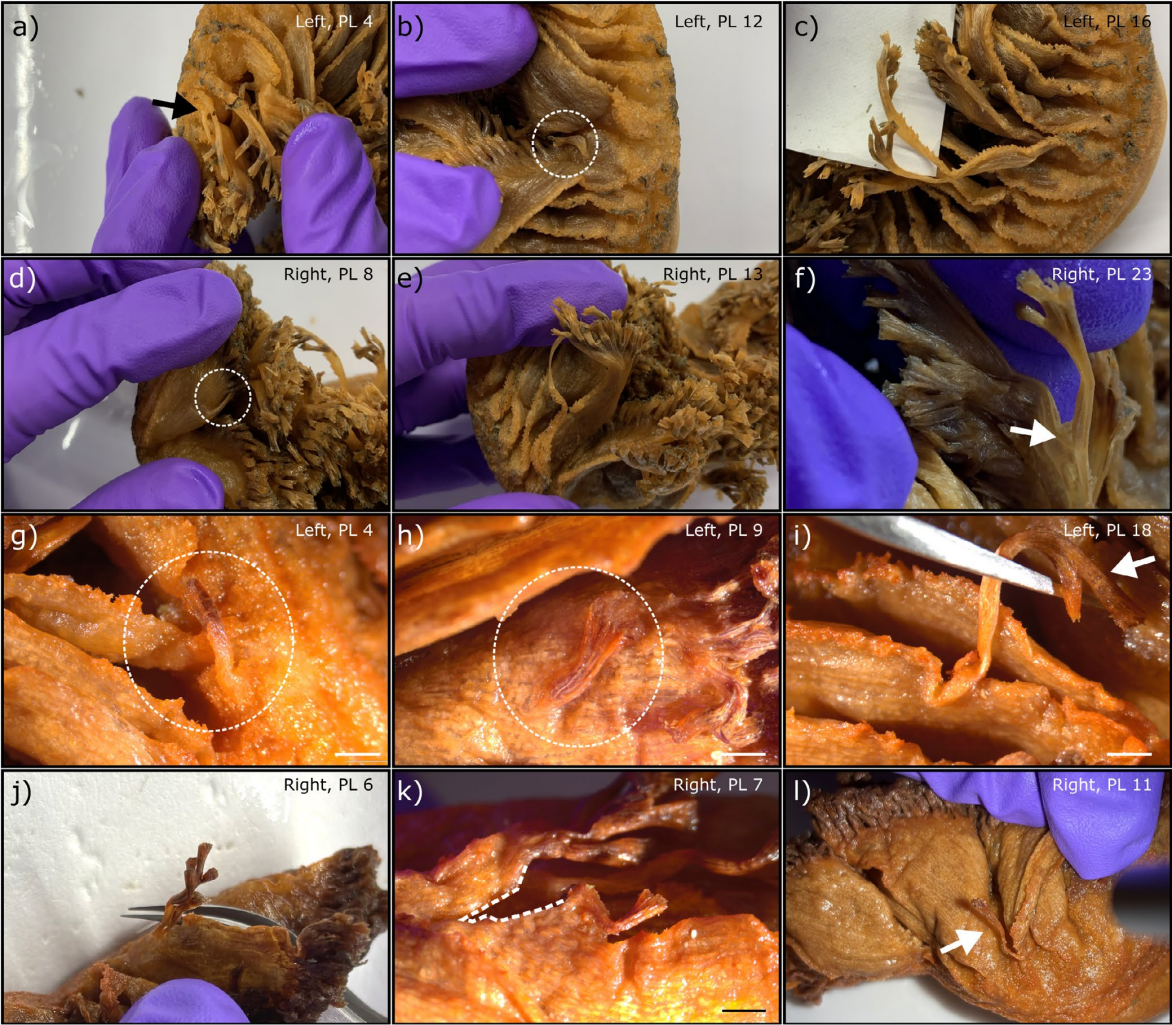
Close‐up of atypical features found on colonies of *Ptilella grayi* (sample 14706 in (a–f), sample 15934 in (g–l)). Detailed in Table 1. Scale bars represent 2 mm.

Colony 14706 is 44.5 cm in total length, with a peduncle 11.5 cm in length and 37 polyp leaves on each side (Figure 5). Colony 15934 is 50.5 cm in total length, with a peduncle 13.5 cm in length and 44 polyp leaves on each side.

#### 3.4.2 Polyp Hypertrophy

Hypertrophied polyps branched from the base of polyp leaves. On colony 14706, the first half of polyp leaf R13 (starting from rachis) has elements of a typical leaf; then halfway through, a thin “branch” with only two autozooids separates from the main leaf (Figure 5E). Mesozooids are seen on both parts. The base of polyp leaf L16 also divides into two separate portions, but with mesozooids present only on the thin branch and being absent on the main part of the leaf (Figure 5C). Polyp leaves in this colony are thinner than those observed in colony R20‐7. Hypertrophied polyps in this colony ranged between 28 and 48 mm in length.

On colony 15934, hypertrophied polyps branched from the margin of the polyp leaves in two occurrences. The first on L18 where 2 cm from the base of the polyp leaf, a hypertrophied “branch” begins and terminates into two polyps (Figure 5I). The second on R11, where a single hypertrophied polyp branches from the margin of the polyp leaf (Figure 5L). Hypertrophied polyps in this colony ranged between 10 and 15 mm.

#### 3.4.3 Polyp Leaf Split

Polyp leaf splits were recorded on L5, L6, R21, and R23 of colony 14706, and R6 and R7 of colony 15934 (Figure 5).

#### 3.4.4 Autozooid Budding on Polyp Leaves

Autozooid budding was observed on polyp leaves L12 and R8, R22 of colony 14706 (Figure 5). In this colony, polyp leaf L4 appears to be a modified or atrophied polyp leaf, with a few mesozooids and only two autozooids, contrary to neighboring polyp leaves possessing dozens of autozooids (Figure 5A). On colony 15934, a single autozooid bud was observed on L9. Two attached polyps also budded just below the base of L4.

### 3.5 *Pennatula aculeata/Pennatula* sp.

#### 3.5.1 General Morphology

Colony lengths ranged between 21.0 and 30.5 cm, with peduncle length ranging between 8.2 and 10.7 cm. Three colonies were partially missing their peduncles and could not be measured. Each side of the rachis did not always have the same number of polyp leaves but was similar (within a few polyp leaves of each other, four maximum). The number of polyp leaves between colonies ranged between 32 and 49.

#### 3.5.2 Polyp Hypertrophy and Polyp Leaf Split

**FIGURE 6:**
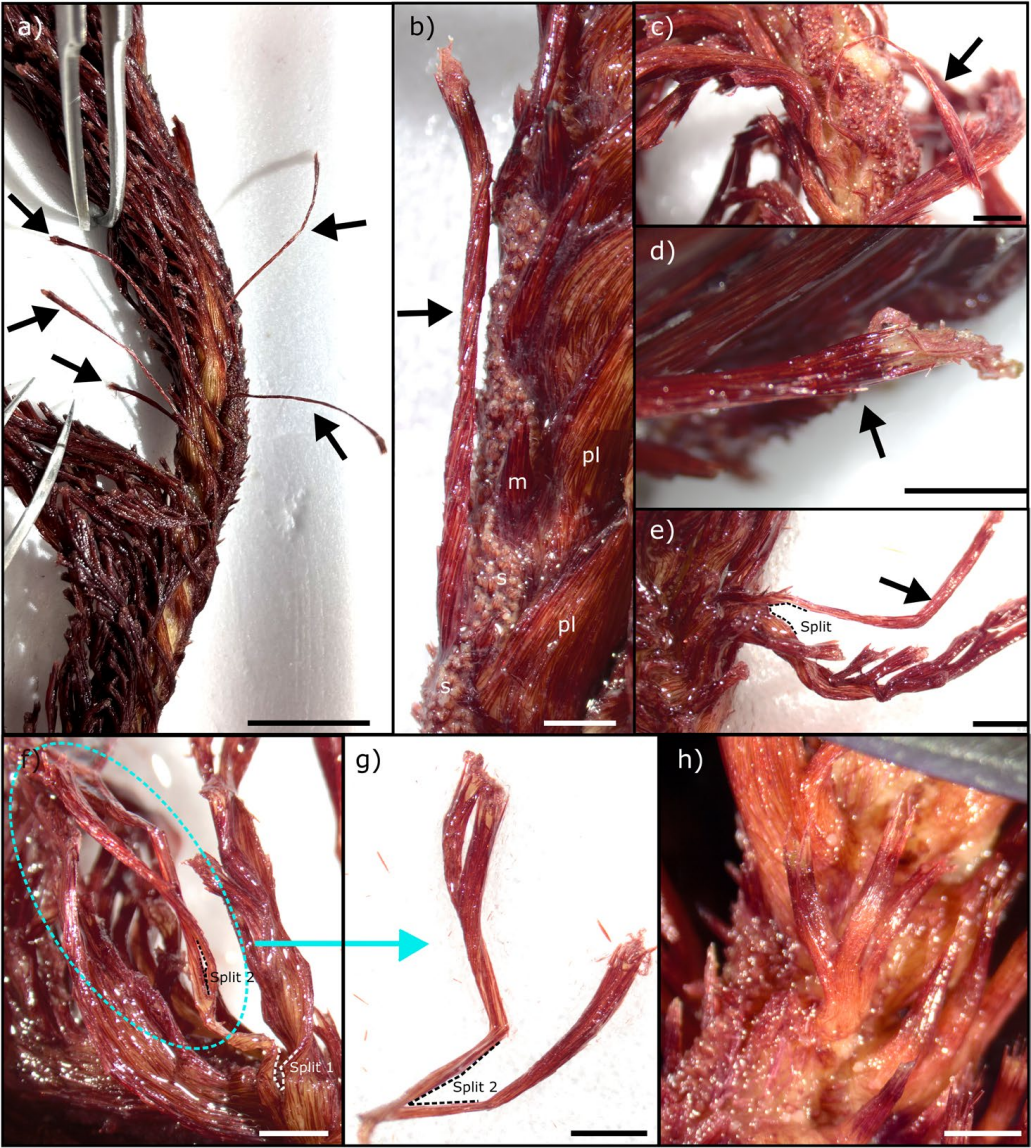
Close‐up of atypical features found on colonies of *Pennatula aculeata* or *Pennatula* sp. including hypertrophied polyps (a–d), split leaves with hypertrophied polyps (e–g) and atrophied leaves (h). m, mesozooid; pl, polyp leaf; s, siphonozooids. Arrows point to hypertrophied polyps. Blue circle indicates location of polyps shown in (h). Scale bars represent 2 cm in (a) and 2 mm in (b–h).

The number of hypertrophied polyps per colony ranged between 1 and 24. In most occurrences (*N* = 45 polyps) hypertrophied polyps grew on the dorsal side of the rachis adjacent but not necessarily connected to the corresponding polyp leaf, among the siphonozooids and mesozooids (Figure 6A–C). These hypertrophied polyps ranged between 9 and 35 mm in length, a few times reaching lengths close to that of the adjacent polyp leaf. The hypertrophied polyps were densely packed with sclerites, like a typical *Pennatula* autozooid (Figure 6D).

In two colonies (colonies 15791 and 15942‐2), the hypertrophied polyps arose from branching from polyp leaves. In colony 15791, one hypertrophied polyp split from the top of polyp leaf R16 (~2 mm from the base of the leaf) and appears to have grown parallel to it (Figure 6E). In the same colony, another leaf (R6) first split into two, similar to R16 (Figure 6F), with hypertrophied polyps splitting away from the rest of the polyp leaf that contained the autozooids. But in this occurrence, one of the halves further split and produced two parts: a single hypertrophied polyp (~10 mm), and a second part terminating into two polyps (~13 mm length of longest polyp) (Figure 6G). In the second colony (15942‐2), the hypertrophied polyp (13 mm length) split from the polyp leaf ~0.3 mm from the polyp leaf base.

#### 3.5.3 Autozooid Budding on Polyp Leaves

No autozooid budding was observed on the polyp leaves of *P. aculeata* or *Pennatula* sp.; however, colony 15942‐2 had two modified or atrophied polyp leaves, one with 3 polyps on the leaf (R6, Figure 6H) and the other with 9 (R7).

## 4 Discussion

**FIGURE 7:**
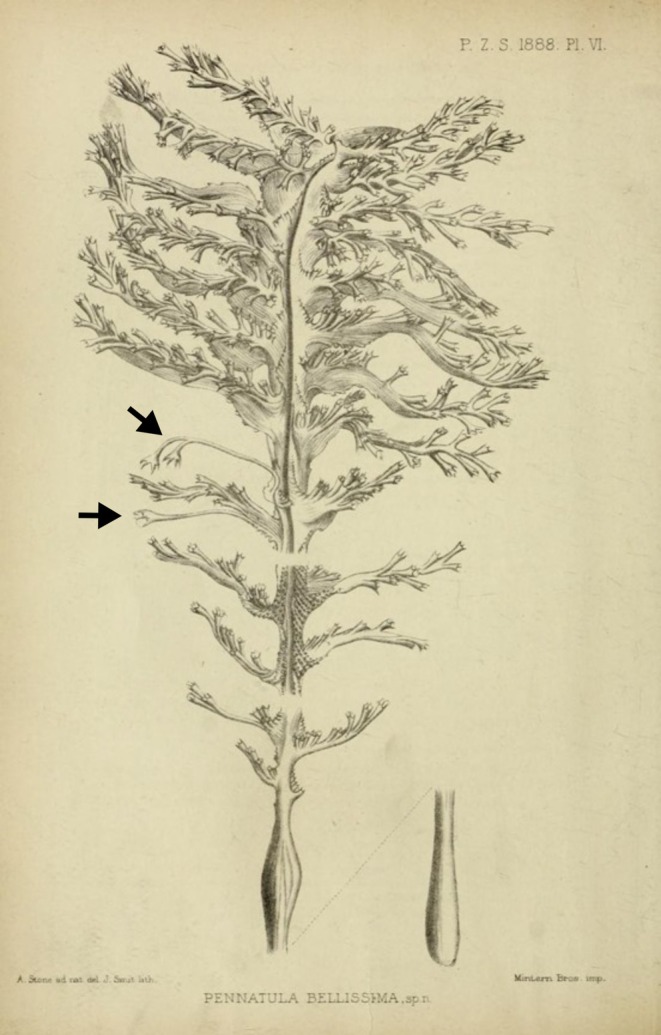
Sketch of *Ptilella bellissima*'s holotype as published by Fowler (1888) as *Pennatula bellissima*, with arrows (added here) pointing to hypertrophied polyps similar to those reported in this study.

To our knowledge, the observations revealed here have not been previously reported as species traits, except for a note brought up by Fowler (1888) when describing *Ptilella bellissima*'s holotype (published as *Pennatula bellissima*). Fowler (1888) revealed that “At two points easily recognizable on the left‐hand side of plate VI., parts of two leaves have apparently been nibbled away, producing a marked hypertrophy of the remaining polyps”. The *Pt. bellissima* sketch provided by Fowler (1888) (and here reproduced with permission in Figure 7) clearly indicates a similarity to our finding, in particular to Figure 4D (ending with two autozooids). A photograph of the holotype is also displayed in García‐Cárdenas et al. (2019), and the specimen has been deposited in the UK'S Natural History Museum.

Our observations across multiple species and their broad geographic distribution (Figure 1) led us to conclude that these traits have likely been overlooked and unreported and might be more common than initially thought. We have examined some of the classic sea pen literature (e.g., Hickson 1916; Williams 1995) and found no reference to the peculiarities described here. When describing acrozooids, a fifth polyp type found in *Pteroides* spp., Williams et al. (2012) recognized how rare those polyps were after examining several colonies stored in different scientific collections and not finding them. Acrozooids were hypothesized to represent asexual polyps (Williams et al. 2012), therefore with a reproductive function, which could explain their rarity (i.e., might develop under special circumstances or seasonally). Although most of our specimens were collected by bottom trawl, we do not believe that our observations are related to mechanical damage from the trawling process. The healed and undamaged appearance of split polyp leaves and hypertrophied polyps, their conspicuous occurrence on an ROV‐collected specimen, in situ evidence, and presence across taxa, years, sites, and depths point to biological processes. We offer two main hypotheses on the origin of the traits described here: (1) injury/predation, and (2) polymorphism.

Although Fowler (1888) did not specifically use the term predation, the author did suggest that the polyp hypertrophy observed in *P. bellissima* is likely a consequence of polyp leaves having been “nibbled away”. The split could be the result of predation at some point in the life of the sea pen. Sea pens can be a prey item (sometimes a significant one) for sea stars (Mauzey et al. 1968; Birkeland 1974; Kastendiek 1976; Gale et al. 2013), nudibranchs (Birkeland 1974; Kastendiek 1976; Wyeth and Willows 2006; García‐Matucheski and Muniain 2011), and fish (Kastendiek 1976; Davis et al. 1982). The scale worm *N. acanellae*, which was found with the ROV‐collected colony (*Pt. grayi*, R20‐7), has been previously reported in association with *Pt. grandis* in the same region (Hamel et al. 2015). These authors observed pieces of *Pt. grandis*' tissue and sclerites as stomach contents of the scale worm. Although Taboada et al. (2021) suggested that individuals of *N. acanellae* living on the bamboo coral *Acanella arbuscula* are likely feeding on zooplankton, Hamel et al.'s finding shows that the scale worm can ingest sea pen material. It is however unknown whether sea pen tissue is a common item in this scale worm's diet. The diet of Polynoidae scale worms is known to also include items captured accidentally when attempting to feed on specific prey (Jumars et al. 2015).

Whether predation by this scale worm or another animal could be at least partly responsible for some of the morphological traits identified in this study (especially the polyp leaf splits) is still unconfirmed. Octocorals exposed to predation can show modifications in their morphology. For instance, Benayahu (1998) suggested that predation of the soft coral *Sinularia nanolobata* influenced colony lobe morphology. Soft coral bite marks as a response to intense fish predation have been reported by Garra et al. (2020). These authors found that in some cases bite marks healed within 31 days and completely regenerated the lost tissue 417 days after bites.

We found scarce information detailing morphological changes associated with sea pen predation, including bites that remove entire portions of a sea pen, as observed in *Stylatula elongata* (Davis et al. 1982). Birkeland (1974) showed that the sea star *Mediaster aequalis* digs up the sea pen *Ptilosarcus gurneyi* and eats it “like corn‐on‐the‐cob”, rotating it as it brings it up. There are multiple references to bites from nudibranchs in the study by Birkeland (1974). Even so, reports of predation focus on behavior and mortality/population ecology. The consequences of predation/predation attempts on the surviving colonies, including the impact of injuries and healing mechanisms, are less well known.

Studies on the regeneration and cicatrization (healing) potential of sea pens are rare, but a high regenerative capacity was identified in the genus *Renilla*, which can even regenerate a lost peduncle (Wilson 1903). Pérez and Zamponi (2000) reported two peduncles in a single *Renilla muelleri* colony from Brazil. On the other hand, regeneration capacity from induced injury in *Veretillum cynomorium* was deemed undeveloped, despite a high cicatrization capacity (Tuzet and Paris 1963). These two sea pens do not have polyp leaves and to our knowledge no studies have assessed injury and cicatrization/regeneration in sea pens with polyp leaves, such as the sea pens included in this study. In a detailed octocoral regeneration study, Nadir et al. (2023) found that *Xenia umbellata* regenerated different body parts in days and that amputated tentacles developed into new polyps within a few weeks. There was no indication of regenerated autozooids displaying a different morphology than the other autozooids (*Xenia umbellata* is monomorphic). These authors showed that a smooth tissue was formed over an amputated region of the polyp after injury (i.e., amputation), and that the wounded area appeared closed 2 days after amputation, attesting to a high cicatrization and regeneration capacity in this species. The predation hypothesis remains a possibility, but studies on how these sea pens recover following injury would be required to corroborate or refute it.

Our second hypothesis is related to colony polymorphism. Although oozoids, autozooids, and siphonozooids are present in all sea pens, mesozooids are only known in the genera *Pennatula*, *Pteroides*, *Renilla* (Williams 1995; Williams et al. 2012) and *Ptilella* (García‐Cárdenas et al. 2019). The fact that the species depicted here are some of the few sea pen species with a fourth polyp type (i.e., mesozooids) indicates a potentially higher level of morphological plasticity than other sea pen taxa. The hypertrophied polyps are comparable to autozooids in their shape, but since we did not dissect them to avoid destructive sampling of rare specimens and due to their preservation mode (i.e., not appropriate for histology), we cannot compare polyp anatomy and function. We can only hypothesize that hypertrophied polyps could be distinct from autozooids.

In the case of *P. aculeata* /*Pennatula* sp. the hypertrophied polyps most often are observed emerging from the dorsal track, usually adjacent to a polyp leaf, among the siphonozooids and mesozooids. Loentgen et al. (2025) documented the ability of siphonozooids to be precursors to autozooids in the red coral *Corallium rubrum* . Although it is currently unknown whether siphonozooids or mesozooids transition to other polyp types in sea pens, this finding further reinforces the high morphological plasticity observed in octocorals. It remains a possibility that the hypertrophied polyps described here also originate from existing polyps.

Harvell (1994) discusses polymorphism in marine invertebrates and brings up the notion of “helpful monsters”, which are defined as mutants “populated with several novelties that never appear in normal colonies”. Given the inconsistency in the number and location as well as the intra and intercolony rarity of the traits described here, it is possible that our observations represent such novelties. This could provide a basis for new morphological traits if favored by natural selection. We recognize that our study did not affirm that predation is related to the oddities described here, that hypertrophied polyps represent a distinct polyp type, originated from an existing polyp, or that they represent natural selection novelties in sea pens. Rather, our study provides a basis for further studies, which are necessary to corroborate or refute the hypotheses discussed here.

Finally, a different kind of polyp hypertrophy has been reported in octocorals as a result of endoparasitic associations. For instance, endoparasitic copepods can significantly increase polyp size in *Callogorgia* sp. (e.g., Cairns 2010). However, our observations show polyps that have grown in length, as opposed to diameter. Inspection of the hypertrophied polyps did not yield indications of endoparasites, although three unidentified parasitic copepods were observed inside of three regular autozooids in colony R20‐7 (B. M. Neves, pers. observation). Therefore, we have no evidence to suggest that hypertrophied polyps are related to parasitic associations.

## 5 Conclusions

The novel record of morphological features observed in the sea pens included in this study brings to light questions regarding the mechanisms behind regeneration, cicatrization, polymorphism, and the origin of novelties that could eventually lead to speciation in these taxa. The potential functional role of the hypertrophied polyps is of interest for further study, as we showed that our observations are not lone occurrences, though the frequency in which they appear remains unknown. As such, the potential links between the hypertrophied polyps, split polyp leaves, and polyp budding on the surface of polyp leaves, are presently undetermined. We urge that if colonies with similar traits are found, that these are reported and tissue samples appropriately preserved for anatomical and histological analyses, and potentially RNA (e.g., transcriptomics) for a better understanding of their origins, potential functions in the colony, and comparison to autozooids, particularly the hypertrophied polyps.

## Author Contributions

**Bárbara de Moura Neves:** conceptualization (lead), data curation (lead), formal analysis (lead), funding acquisition (lead), investigation (lead), methodology (lead), project administration (lead), resources (lead), supervision (lead), validation (lead), visualization (lead), writing – original draft (lead), writing – review and editing (lead). **Kathryn Murray:** data curation (equal), investigation (equal), validation (equal), visualization (equal), writing – review and editing (equal). **Rachelle Dove:** data curation (equal), investigation (supporting), methodology (supporting), validation (supporting), writing – review and editing (equal). **Vonda E. Hayes:** data curation (equal), investigation (supporting), resources (equal), writing – review and editing (equal). **Carlos Daniel Pérez:** conceptualization (supporting), writing – review and editing (equal).

## Funding

This work was supported by Fisheries and Oceans Canada. Coordenação de Aperfeiçoamento de Pessoal de Nível Superior (CAPES) (Code 001). National Council for Scientific and Technological Development #315253/2023‐1.

## Conflicts of Interest

The authors declare no conflicts of interest.

## Data Availability

All data associated with this manuscript is provided in the manuscript. Raw specimen photos can be made available upon request.
